# Evidence-based treatment of leprosy and its reactions

**DOI:** 10.1016/j.jdcr.2024.08.048

**Published:** 2024-10-01

**Authors:** Ronald Pust

**Affiliations:** Professor Emeritus, University of Arizona, College of Medicine, Tucson, Arizona

**Keywords:** Hansen’s Disease, leprosy, leprosy reaction, leprosy treatment

*To the Editor:* All 3 of the case reports about leprosy (Hansen’s Disease) published in the past 6 months 1, 2, 3 are valuable in educating clinicians in and beyond dermatology about leprosy in the United States.

Reference 1, a case in New York City, was treated with an evidence based, internationally accepted regimen for active leprosy and for erythema nodosum leprosum.

Reference Cases 2 and 3 were treated with a regimen of once-monthly rifampin, moxifloxacin, and minocycline. This regimen has not been evaluated in any carefully designed clinical trial and its safety and efficacy have not been determined. It is not recommended by the World Health Organization, the Centers for Disease Control and Prevention, or the International Federation of Leprosy Associations.

Leprosy is a serious infectious disease for which a standardized and universally recommended multiple drug treatment has proven safe and effective. While improved regimens are desirable,^4^ a medically conservative, evidence-based approach would advise caution in using the rifampin, moxifloxacin, and minocycline regimen until data from well-designed clinical trials -- comparing rifampin, moxifloxacin, and minocycline directly to standard multiple drug treatment -- show that its safety and efficacy are at least equivalent to multiple drug treatment. Evidence regarding safety and efficacy would include data on the incidence and severity of leprosy reactions and the development of drug-resistant organisms. This approach would be comparable to what is done to develop new treatment regimens for other serious infectious diseases such as tuberculosis.

## Conflicts of interest

None disclosed.
